# Supplementary material for: Methyl Jasmonate Induces Genes Involved in Linalool Accumulation and Increases the Content of Phenolics in Two Iranian Coriander (Coriandrum sativum L.) Ecotypes
Source: Genes (Basel). 2022 Sep 24;13(10):1717. doi: 10.3390/genes13101717 (PMC9602312; doi:10.3390/genes13101717)
Supplement: Supplementary file 1 [file genes-13-01717-s001.zip › genes-1906720-supplementary.pdf]

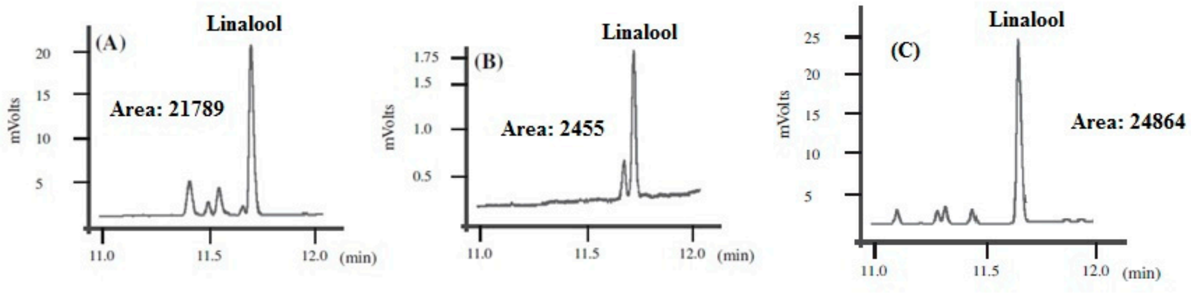

Figure S1. Gas chromatogram for (A) linalool standard (ranging from 12,285 to 220,604 µg/L) (B) CsLIN product in control and (C) CsLIN product in 150 µM MeJA treated-plants.

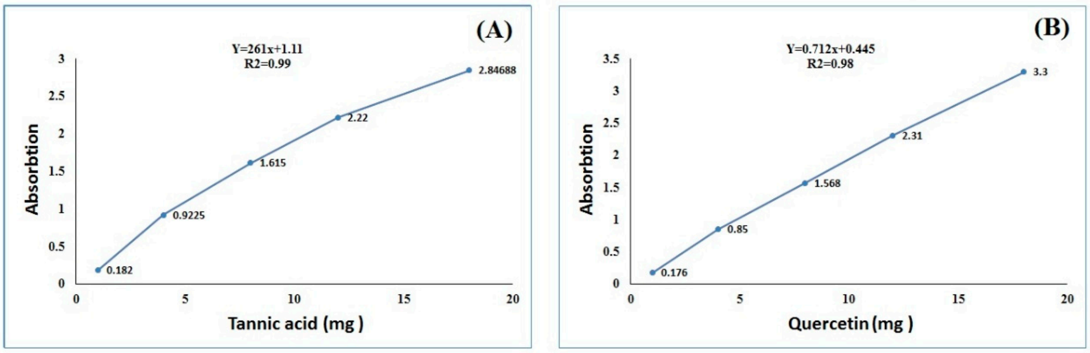

Figure S2. Standard curve of total phenolic content (A) and total flavonoid content (B).

Table S1- Essential oil (EO) terpene composition of control plant. Error bar are in percent standard deviation.

Composition	% of total
(S)-linalool	55.44 ± 1.97
Cymene	13.52 ± 1.65
Ocimene	9.27 ± 2.44
Camphor	7.38 ± 1.29
γ-Terpinene	5.22 ± 1.55
Limonene	3.88 ± 0.1
Linalool oxide	3.14 ± 0.18
Geraniol	0.65 ± 0.24
β-Phellandrene	0.52 ± 0.12
Sabinene	0.45 ± 0.16
Camphene	0.35 ± 0.12
Terpinen-4-ol	0.21 ± 0.1
Borneol	0.21 ± 0.05
α -Terpineol	0.20 ± 0.08
Terpinolene	0.11 ± 0.02
